# Psychometric properties of the Chinese version of the pediatric quality of life inventory 4.0 Generic core scales among children with short stature

**DOI:** 10.1186/1477-7525-11-87

**Published:** 2013-05-30

**Authors:** Hua-hong Wu, Hui Li, Qian Gao

**Affiliations:** 1 Department of growth and development, Capital Institute of Pediatrics, Ya-Bao Road, Chao-Yang District, Beijing, China; 2 Maternal and Child Care Service Centre of Chao-Yang district, Hua-Wei Road, Chao-Yang District, Beijing, China

## Abstract

**Background:**

The quality of life in children with short stature was rarely studied in China, so we explore these children’s quality of life and psychometric properties of the Chinese version of the Pediatric Quality of Life Inventory 4.0(PedsQL4.0) Generic Core Scales among children with short stature.

**Methods:**

A total of 201 children aged 8 ~ 18 years from the short stature clinic and other clinics of capital institute of pediatrics attended this study. The questionnaires include demographic information and PedsQL4.0 generic core scales. According to children’s height, we divided them into three groups: short stature, normal short and normal group, then compared the score of scales by the height category. Moreover, we analyzed the reliability and validity of PedsQL4.0 generic core scales in these 201 children.

**Results:**

The child self-report total PedsQL mean score, for the short stature, normal short and normal groups were 77.77 ± 9.69, 83.50 ± 8.56 and 87.36 ± 7.23; the parent-proxy total PedsQL mean score were 77.62 ± 10.50, 82.69 ± 8.35 and 84.91 ± 9.96 respectively. Both for children self- and parent proxy-reports, the Cronbach’s α coefficients of total scale, psychosocial health and social functioning ranged between 0.74 and 0.80, it ranged between 0.51 and 0.66 in other dimensions. For child self-reports, the correlation coefficients of 17 items’ scores (total 23 items) with the scores of dimensions they belong to were above 0.5, with the highest 0.759; the other 6 items’ correlation coefficients were below 0.5, with the lowest 0.280. For parent proxy-reports, the correlation coefficients of 19 items’ scores with the scores of dimension they belong to were above 0.5, with the highest 0.793, the other 4 items’ below 0.5 with the lowest 0.243.

**Conclusions:**

The quality of life in children with short stature is worse than their normal peers by Peds QL4.0 generic core scales, the statues of their quality of life was positively related to their stature.

## Background

Quality of life (Qol)was defined as people’s experience on status of life related to their goals, expectation, standards and those things they care about, and it varies in different cultures and value systems [1]. In China, according to the quick development of economy during the past decades, people started to pay more attention on their Qol, short stature meanwhile became to be a reason for complains in families, schools and clinics. Varni, Stephen and Limbers has reported that pediatric patients with short stature experienced bad school functioning, cognitive functioning and impaired quality of life than healthy children [2,3]. The limitations of body and immature looks caused by short stature lead these children to be treated differently and unconsciously discriminated by peers and adults [4]. Short stature may influence their choices of work and marriage after grow up [5]. If not been treated, they may suffer from those problems throughout their life. So, in order to identify children at increased risk for impaired Qol and intervene timely, we need an appropriate scale to evaluate the Qol of these children.

Since last century, many studies have focused on pediatric Qol in order to improve patients’ health and status of life and evaluated the value of health care. The pediatric quality of life inventory measurement models (Peds QL) was one of the most popular scale systems for children’s Qol evaluation [6]. There were some studies have lend support to the use of the PedsQL 4.0 Generic Score Scales in routine assessment of children with short stature. However, there was no study on evaluation of short children’s life status applying the Peds QL4.0 generic core scale in China. So we firstly used the Chinese version of Peds QL4.0 generic core scale to evaluate the Qol of children with short stature, and explored the psychometric properties of this scale among them.

## Methods

### Questionnaire and scales

Questionnaire consisted of the demographic information of children such as gender, age, height, parents’ education background, family income and other factors could inflect children’s normal growth such as birth history, disease history, diet habit, academic record, total sleep time.

Chinese Version of the Pediatric Quality of Life Inventory 4.0 Generic Core Scales(Peds QL4.0 scales) was applied to evaluate children’s quality of life. This scale was composed of 23 items which divided into 4 dimensions, physical health (8 items), emotional functioning (5 items), social functioning (5 items) and school functioning (5 items). The latter 3 dimensions can also be united and called psychosocial health. There are child self-report and parent proxy-report.

### Subjects

Children and their parents were recruited from short stature clinic and other clinics in Capital Institution of Pediatric in China. There were 201 children (aged 8 ~ to 18 years, 111 boys and 90 girls) and their parents attended this survey, the sex ratio was 1.23/1. Those children were divided into three height category by the Chinese children and adolescent growth standard published in 2009 [7], short stature group (97 children) defined as height blew the 3th percentile of the same sex and age children, normal short group (69 children) as height between the 3th and 25th percentile, normal group (35 children) as height above the 25th percentile. The short stature group was wildly recognized as pathologic, including idiopathic short stature 46.39%, growth hormone deficiency 36.08%, small for gestational age 5.15%, turner syndrome 5.15% and others 7.22%.

### Data collecting

Children’s height was measured by trained nurses and doctors. The questionnaire and PedsQL4.0 scale were all interview-administered one by one. The child self-reports were filled solely on children’s answers and parent proxy-reports e on the answers of parents.

### Statistics

The data were analyzed by SPSS 13.0 software. Continuous variables were described by mean ± SD, the PedsQL 4.0 Generic Core Scales scores’ differences among short stature, normal short stature and normal group were computed using con-variable test, differences between HtSDS < −3 and −3 < HtSDS < −2 group using independent sample *t*-tests. *P* <0.05 was considered statistically significant. The reliability and validity of Peds QL4.0 scales in these 201 children were calculated by Cronbach’s Θ coefficients and Pearson correlation coefficients. In general, a Cronbach’s Θ coefficient ranging from 0.70 to 0.84 is regarded as satisfactory inter-consistence. Pearson’s Correlation coefficient effect sizes are designated as small (0.10 to 0.29), medium (0.30 to 0.49), and large (0.50 or more) in magnitude.

### Ethical approval

This study was approved by Ethics Committee of Capital institute of pediatrics.

## Results

A total of 201 children aged 8 ~ 18 years participated in this survey; the male to female ratio was 1.23. The child self-report of Peds QL4.0 scales were all completed and 2 of parent proxy-reports were not completed because those two parents worked away from home and don’t know children’s everyday life well. The time spend to finish scales was not long, child self-reports 4.29 ± 1.38 minutes and parent proxy-reports 5.02 ± 1.33 minutes.

### The Qol of 201 children

The total scale scores and scores of every dimensions were showed in Table 1. The differences among short stature, normal short and normal groups were also explored. In child self-report, there were all significant differences on the scores of total scale and every dimensions among this three groups (*p* < 0.05); in parent proxy-report, there were also significant differences on the scores of total scale and most dimension (*p* < 0.05), except for social functioning. The scores of short stature group were lower than normal short and normal groups, the normal group got the highest score.

**Table 1:** The scores of peds QL4.0 core scales in three groups

	**NO. of item**	**Short stature group (n = 97)**	**Normal short group (n = 69)**	**Normal group (n = 35)**	***p***
		**mean ± SD**	**mean ± SD**	**mean ± SD**	
Self-report scale					
Total scale	23	77.77 ± 9.69	83.50 ± 8.56	87.36 ± 7.23	0.000
Physical health	8	80.09 ± 9.19	87.32 ± 7.97	90.71 ± 7.97	0.000
psychosocial health	15	74.93 ± 11.62	81.50 ± 10.98	85.57 ± 7.80	0.000
Emotional functioning	5	69.79 ± 13.69	73.99 ± 17.42	79.71 ± 14.29	0.009
Social functioning	5	82.58 ± 17.23	91.25 ± 12.47	95.71 ± 7.19	0.006
School functioning	5	72.42 ± 15.16	79.38 ± 17.51	81.29 ± 11.9	0.006
Parent proxy-report scale					
Total scale	23	77.62 ± 10.50	82.69 ± 8.35	84.91 ± 9.96	0.001
Physical health	8	85.39 ± 11.88	88.24 ± 9.93	92.23 ± 8.27	0.009
psychosocial health	5	67.16 ± 13.98	72.50 ± 16.47	76.52 ± 18.35	0.010
Emotional functioning	15	73.47 ± 11.53	79.73 ± 10.40	81.01 ± 12.82	0.000
Social functioning	5	84.21 ± 18.02	89.49 ± 14.46	91.06 ± 11.97	0.072
School functioning	5	69.05 ± 16.26	77.21 ± 14.15	75.45 ± 16.32	0.024

Then we split the short stature group into two parts by height Standard Deviation Score (HtSDS), HtSDS < −3 and −3 < HtSDS < −2, and compared the status of Qol between them. Table 2 showed us that score differences existed in total scale, psychosocial health, social functioning and school functioning (*p* < 0.05) but not in physical health and emotional functioning. The Qol of −3 < HtSDS < −2 group was better than HtSDS < −3 group.

**Table 2:** The scores of peds QL4.0 core scales in two HtSDS level groups

	**HtSDS < −3 group (n = 27)**	**−3 < HtSDS < −2 group (n = 62)**	***p***
	**mean ± SD**	**mean ± SD**	
Self-report scale			
Total scale	74.52 ± 10.82	79.40 ± 9.14	0.031
Physical health	80.17 ± 10.64	84.07 ± 8.35	0.125
psychosocial health	71.17 ± 12.71	76.91 ± 11.05	0.034
Emotional functioning	69.44 ± 13.11	70.48 ± 13.78	0.741
Social functioning	75.56 ±17.56	84.60 ± 17.02	0.025
School functioning	68.52 ±18.49	75.65 ± 13.74	0.047
Parent proxy-report scale			
Total scale	75.44 ± 12.22	79.17 ± 10.09	0.140
Physical health	83.68 ± 12.33	86.41 ±12.09	0.336
psychosocial health	71.05 ± 13.95	75.31 ±10.62	0.122
Emotional functioning	67.59 ± 13.82	69.00 ±13.86	0.662
Social functioning	79.81 ± 19.14	85.42 ±17.50	0.183
School functioning	65.74 ± 21.96	71.50 ±13.57	0.138

The correlation analysis showed that children’s status of Qol is positively related to their HtSDS (*r*_*s*_ =0.436,*p* < 0.01) and academic record (*r*_*s*_ =0.218,*p* < 0.01). There were no correlations between age, gender, parents’ education background, family income and children’s Qol.

### The reliability of Peds QL 4.0 scales

Table 3 showed us the inter consistencies of Peds QL 4.0 scales in total scale and every dimensions. For child self-report and parent proxy-report, the Cronbach’s α coefficients of total scale, psychosocial health and social functioning were from 0.74 to 0.80 which supported high reliability. Whereas the other dimensions’ Cronbach’s α coefficients ranged from 0.51 to 0.66 gave a hint of lower reliability.

**Table 3:** The cronbach’s α coefficients of peds QL4.0 core scale

**Dimension**	**Self-report scale**	**Parent proxy-report scale**
Total scale	0.80	0.80
Physical health	0.51	0.66
psychosocial health	0.76	0.75
Emotional functioning	0.55	0.53
Social functioning	0.74	0.79
School functioning	0.55	0.60

### The validity of Peds QL 4.0 scales

We explored the correlations between scores of every item and the scores of that dimension they belong to. For child self-report, 17 of 23 items’ Pearson coefficients are above 0.5, with the biggest 0.759; the other 6 items blew 0.5, with the smallest 0.280, all these correlation coefficients are statistically significant. For parent proxy-report, 19 of 23 items’ Pearson coefficients are above 0.5, with the biggest 0.793; the other 4items blew 0.5, with the smallest 0.243. These 4 items all belonged to physical health dimension.

## Discussion

Peds QL scales was firstly studied by varni and his colleagues in San Diego children hospital and health center in 1987, now they include a general core scale and other eight diseases models [6]. These scales meet the needs proposed by world health organization that Qol should refer to physical, psychological health, social and role functioning, so have been widely used around the world. Moreover, the items selected for the latest version of Peds QL 4.0 general core scale can reflect the universal concern in children and adolescents aged 8 ~ 18 years. The state of California has selected the Peds QL 4.0 general core scale for the health outcomes evaluation of the state’s health insurance program [8]. In 2004, this scale system was introduced into China after a translation-reverse translation-culture adaptation-pre-experiment procedure [9].

### The Qol of children with short stature

This is the first time that Chinese version of Peds QL 4.0 general core scale has been used to evaluate the Qol of short children. So far, Peds QL 4.0 general scale has been translated into 53 languages [10-12], and widely used in health or chronic disease children [13,14]. But we have not found any study on evaluating Qol of short stature children by this scale in China. Recently, more and more Chinese children see doctors because of their height problems. They may have suffered from a series of physical, social and psychological problems [4]. So an appropriate scale to score their status of Qol was needed.

The Qol of children with short stature was worse than their normal peers, and the statues of their Qol was positively related to their stature. For child self-report, the mean score of total scale in normal group was 87.36 points which is almost no difference from the results of other study [15]. The normal short group scored 83.50, short stature group 77.77 which was 9.59 points lower than normal group. For parent proxy-report, the scores’differences among these three groups were all statistically significant except the scores of social functioning. This may due to the discrepancy of parents’ and children’s own awareness of their social interaction. Furthermore, as to the two subgroups of short stature group, HtSDS < −3 group scored lower than −3 < HtSDS < −2 group about 5 points in every dimension, which concluded the status of Qol correlated to the stage of disease [6,16]. Besides, some studies showed cardiopulmonary function of patients with growth hormone deficiency is abnormal and can influence patients’ physical function [17], but this view was not been proved in this study.

The Qol of children with short stature is not as well as normal children but meanly better than children with other chronic diseases. The individual variability in Qol among different patients was huge, the Qol can be normal or badly damaged, so individual assessment is absolutely necessary. In 2005 and 2007, varni and his colleagues explored the Qol of normal children and pediatric patients with ten kinds of chronic diseases (including diabetes, mental illness, heart disease, asthma, obesity, end-stage renal disease, gastrointestinal disease, cancer, rheumatic diseases and cerebral palsy) using Peds QL 4.0 general core scales, normal children gain the highest score 82.7. Among these 10 diseases groups, diabetes scored highest 76.62 and cerebral the lowest 51.28 [15,18]. In our study, the mean score of short stature group (77.77) was close to the diabetes group and obviously higher than cerebral palsy mentioned above. However, there was also one short children get 44.57 points which even lower than the cerebral palsy group’s mean value. So we stress again the individual assessment.

### The reliability and validity of Peds QL 4.0 scales

For both child self- and parent proxy-report, the Cronbach’s α coefficients of physical health, emotional functioning and school functioning were blow 0.7, which suggested lower inter consistencies, the Cronbach’s α coefficients of total scale and other two dimensions were bigger than 0.7. A survey on 3716 school children in Guang Zhou, China, in 2008 showed the total scale’s α coefficients was 0.9 which is close to the result of varni(α = 0.92) [19]. Another survey on 335 children with cancer in Hong Kong 2012 also showed better reliability, total scale’s α coefficient bigger than 0.9 and all dimensions’ bigger than 0.7 [20]. In our study, total scale’s α coefficient was 0.8. We may need more samples to verify the validity of Peds QL 4.0 general scale on short children.

As to validity, in our study the scores’ correlation coefficients between 74% of items with the dimensions they belong to were bigger than 0.5, suggested a good validity. Other 6 items’ correlation coefficients was blew 0.5, including “It is hard for me to take a bath or shower by myself” which was also mentioned by Chen Yu-ming [14], “It is hard for me to do chores around the house”, “I hurt or ache”, “I have trouble sleeping” and “I miss school to go to the doctor or hospital”. The reason for these 6 items were picking out may be the heavy burden of school work, sleeplessness, lack of exercise, less housework of Chinese children.

### The necessity of Qol evaluation on children with short stature

For most children with short stature, their physical function are almost normal, their therapy is more likely to be a kind of health intervention. And the biggest challenge of health intervention was to identify those patients whose Qol wound be damaged for a long time, then give them appropriate therapy in order of improve their status of Qol. So, applying standardized scale to screen short children who need be intervened timely should emerge as a necessary procedure in clinical practice. Qol evaluation has been widely used in cancer, chronic diseases and special populations, to provide a comprehensive basis for choices of treatment and interventions, decision-making of health resource allocation. As all we know, the more serious damage in children’s Qol the more urgent need for effective and specific therapy [15].

Qol evaluation should be an important part of all these procedures : assessment of the health damage of short stature in children, decision-making of clinical practice and evaluation of therapy result. In other words, clinic doctors should not pay their attention solely on height of children with short stature, but more on their Qol. This is not only for children with short stature, but for all chronic patients. In the rheumatology clinic sample, when the pediatric rheumatologist examined the completed Peds QL instrument at the point of service and made a clinical intervention decision based on the findings, the subsequent child self-report Peds QL4.0 generic core scales were significantly higher approximately by 10 points [8]. Except for the field of diseases, Qol scales also should be a necessary tool to screen children with physical and psychological problems in health care [21].

## Conclusions

In conclusion, it was the first time we used Peds QL4.0 generic core scale to evaluate the Qol of children with short stature, and found the Qol of these children was worse than their normal peers, the statues of their Qol was positively related to their stature. Qol evaluation should be an important part of assessment of the health damage of short stature in children, decision-making of clinical practice and evaluation of therapy result.

## Abbreviations

QOL: Quality of life; PedsQL: Pediatric quality of life inventory.

## Competing interests

The authors declare that they have no competing interests.

## Authors’ contributions

LH conceptualized the rationale and design of the study. WH-H and GQ were in charge of the data collecting. WH-H performed the statistical analyses. LH and WH-H drafted the manuscript. All authors read and approved the final manuscript.
